# A Novel Chimeric Fiber-C4/D11 Subunit Vaccine Induces Cross-Neutralizing Antibodies and Provides Better Protection Against Fowl Adenovirus (FAdV) Type 4 and Type 11 Than the Fiber-D11/C4 Subunit Vaccine

**DOI:** 10.3390/vetsci12090920

**Published:** 2025-09-22

**Authors:** Xiangqin Wang, Kuan Zhao, Baishi Lei, Wenming Jiang, Yanliang Qiao, Wanzhe Yuan

**Affiliations:** 1 College of Veterinary Medicine, Hebei Agricultural University, Baoding 071001, China; 2 National Research Center of Engineering and Technology for Veterinary Biologicals, Nanjing 210014, China; 3 Group Biological Products R and D Center, Shandong Sinder Technology Co., Ltd., Qingdao 266100, China; 4 Laboratory of Surveillance for Avian Diseases, China Animal Health and Epidemiology Center, No. 369 Nanjing Road, Qingdao 266032, China

**Keywords:** FAdV-4, FAdV-11, fiber, chimeric subunit vaccine, protection

## Abstract

The widespread prevalence of different serotypes of fowl adenoviruses (FAdVs) has led to diverse vaccine demands, especially for subunit vaccines targeting FAdV-4 and FAdV-11, which cause hydropericardium-hepatitis syndrome (HHS) and inclusion body hepatitis (IBH), respectively. Although the Fiber protein is known to elicit robust immune protection, further exploration is needed to enhance the production of cross-neutralizing antibodies. This study utilized structural prediction and homology modeling techniques, employing domain-swapping strategy to integrate neutralizing epitope-containing amino acid sequences (274–451aa and 364–543aa) into the shaft domain of the Fiber protein. Two novel chimeric proteins were recombinantly expressed and developed into subunit vaccines: Fiber-C4/D11 and Fiber-D11/C4. Immunogenicity assessments revealed that the Fiber-C4/D11 vaccine group rapidly induced an antibody response against FAdV-11 within 7 days post-vaccination. By 28 days post-vaccination (dpv), the Fiber-C4/D11 vaccine group exhibited significantly higher levels of cross-neutralizing antibodies compared to the Fiber-D11/C4 group (*p* < 0.05). Challenge experiments demonstrated that both vaccines effectively alleviated clinical symptoms and prevented mortality in SPF chickens. Compared to Fiber-D11/C4, Fiber-C4/D11 significantly reduced body weight loss, liver lesions, viral titers in tissues, and viral shedding. Notably, no cross-neutralizing antibodies were detected following FAdV-4 or FAdV-11 infection, indicating a lack of natural cross-protection between the two serotypes. The chimeric vaccine addressed this gap, offering a promising multivalent approach to mitigate FAdV infections and advancing fowl adenoviral subunit vaccine strategies.

## Simple Summary

In recent years, the frequent outbreaks of hydropericardium-hepatitis syndrome (HHS) and inclusion body hepatitis (IBH) caused by fowl adenoviruses (FAdVs) have posed significant challenges to disease prevention and control. Currently, there is a lack of a single subunit vaccine capable of effectively targeting multiple serotypes, particularly in inducing sufficient cross-protective immune responses. To address this issue, this study employed chimeric protein expression technology, swapping the shaft and knob domain amino acid sequences of the Fiber proteins from FAdV-4 and FAdV-11, successfully developing two novel chimeric subunit vaccines. Experimental results demonstrated that the Fiber-C4/D11 subunit vaccine induced high levels of cross-neutralizing antibodies and conferred robust protection against both FAdV-4 and FAdV-11, effectively overcoming the lack of natural cross-protection between these serotypes. This study offers a new strategy for developing broad-spectrum fowl adenovirus vaccines, contributing to disease control with significant practical application value.

## 1. Introduction

Fowl adenovirus (FAdVs) is a globally prevalent pathogen affecting both domestic and wild chickens, causing significant infectious diseases [1]. FAdV infections lead to hepatitis-hydropericardium syndrome (HHS), inclusion body hepatitis (IBH), and gizzard erosion (GE) [2]. Since the initial outbreak of HHS in Islamabad, Pakistan, in 1987, both HHS and IBH have spread extensively across multiple countries [3,4,5] and regions worldwide [6,7]. In China, since 2015, HHS caused by FAdV-4 [8,9] and IBH caused by FAdV-2, -8a/8b, and -11 have been widely reported across multiple provinces [8,10,11]. FAdV infection also induces immunosuppression, increasing susceptibility to secondary infections by other pathogens, which often results in mortality [12,13]. These outbreaks have caused substantial economic losses in the Chinese poultry industry [14]. Vaccines are the primary means of controlling and preventing avian adenovirus diseases. The inactivated FAdV-4 vaccine, which is widely used in China and South Asia, can provide certain protection against hepatitis-hydropericardium syndrome (HHS) [15,16,17]; live attenuated vaccines can induce strong humoral and cellular immune responses [18,19,20], but there are biosafety concerns; some inactivated vaccines (such as those based on FAdV-8a) also have certain cross-protective effects against FAdV-8b and FAdV-11 [21]. However, existing vaccines are still limited in dealing with serotype diversity and newly emerging variants, with incomplete cross-protection and difficulty in rapid iteration. In contrast, subunit vaccines have attracted much attention due to their high safety and ease of design, especially chimeric vaccines based on the fiber protein, which are expected to achieve broader and more effective immune protection [22,23,24].

FAdVs are non-enveloped viruses with icosahedral symmetry and a diameter of approximately 70–90 nm [25,26]. The genome is double-stranded DNA, ranging in size from 43 to 46 kb. FAdVs are classified into five species (FAdV-A to FAdV-E) and 12 serotypes (FAdV-1 to -8a, -8b to -11) [27]. FAdV-4 contains 10 structural proteins, with the penton, hexon, and Fiber proteins being the main components [18,28,29]. FAdV serotypes (FAdV-2, -3, -5, -6, -7, -8a, -8b, -9, -11) have only one Fiber protein, while serotypes 1, 4, and 10 contain two Fiber proteins. The Fiber protein plays a critical role in viral infection and entry [30,31]. The Fiber protein consists of a knob domain, a long axial stem with a repetitive trimeric motif (a shaft domain), and an N-terminal tail [32]. Studies have shown that the Fiber2 protein of FAdV-4 and its chimeric knob domain can effectively induce T-cell immunity and a strong immune response, and the Fiber1/2 knob subunit vaccine has been proven to provide complete protection against FAdV-4 infection in chickens [33,34,35]. The Fiber1/2 knob subunit vaccine has provided complete protection against FAdV-4 [36]. Fiber-2 protein provides complete protection against fowl adenovirus serotype 4 and induces more rapid and robust immune responses than an inactivated oil-emulsion vaccine [22]. While previous studies have focused on the knob domain [37] and N-terminal tail [23], the immunological impact of modifying the Fiber shaft domain through chimerism or replacement have not yet been explored.

The Fiber protein, a critical surface protein of fowl adenovirus (FAdV), has been a focal point of research, particularly due to the neutralizing epitopes located in its knob domain. These epitopes can induce the production of neutralizing antibodies, effectively blocking viral infection. This study employed a domain-swapping strategy based on known and predicted neutralizing epitopes in the knob domain of FAdV Fiber proteins. Specifically, the shaft domain of one Fiber protein was replaced with the knob domain of another, successfully constructing two chimeric subunit vaccines: Fiber-C4/D11 and Fiber-D11/C4. Through immunogenicity assessments and challenge experiments, the immune protective efficacy of these chimeric vaccines was systematically evaluated.

## 2. Materials and Methods

### 2.1. Cell, Viruses, Plasmids and Animals

Leghorn male hepatoma cells (LMH cells) were obtained from Beijing North Institute of Biotechnology and cultured in Dulbecco’s Modified Eagle Medium/Nutrient Mixture F-12 (1:1) medium (Gibco, Grand Island, NY, USA) supplemented with 10% fetal bovine serum (Gibco, Melbourne, VIC, Australia). FAdV-4 strain SDSX and FAdV-11 strain SD (GenBank accession numbers KY636400 and PQ117790, respectively) were propagated and purified on LMH cells. Viral titers were determined using the 50% tissue culture infectious dose (TCID_50_) method. The plasmid pCold I, maintained in our laboratory, was used as an expression vector for the Fiber-C4/D11 and Fiber-D11/C4 proteins. Specific pathogen-free (SPF) chickens were sourced from Beijing Boehringer Ingelheim Technology Co., Ltd. (Beijing, China).

### 2.2. Expression and Purification of Recombinant Proteins

This study integrated protein structure prediction (AlphaFold2 and PyMOL) with homology modeling (SWISS-MODEL) to analyze the structures of FAdV-4 and FAdV-11 Fiber proteins, predicting the optimal chimeric regions of their shaft domain and potential neutralizing epitopes in the knob domain. Complete replacement of the knob domain may disrupt the overall structural integrity of the fiber protein. To ensure that the three-dimensional structure of the chimeric protein is as close as possible to that of the native Fiber protein and to avoid potential conformational changes resulting from the complete replacement of the knob domain on the shaft, this study adopted a partial domain replacement strategy to design and construct two novel chimeric Fiber proteins. Specifically, the fourth to twelfth β-turns (amino acid residues 128–305) of the FAdV-11 Fiber protein were replaced with the knob domain of FAdV-4 Fiber2 (amino acid residues 274–451). Conversely, the third to eleventh β-turns (amino acid residues 92–244) of the FAdV-4 Fiber protein were replaced with the knob domain of FAdV-11 Fiber (amino acid residues 364–543). The resulting chimeric sequences were synthesized (Sangon Biotech, Shanghai, China) and subsequently cloned into the pCold I prokaryotic expression vector to construct recombinant plasmids pCold-Fiber-C4/D11 and pCold-Fiber-D11/C4. The sequences of the chimeric genes and Fiber genes, the corresponding amino acid sequences, and the primer sequences are provided in the Supplementary Materials. These plasmids were transformed into *Escherichia coli* BL21 (DE3) competent cells for protein expression [38]. The bacterial strains were cultured in LB medium at 37 °C until the OD_600_ reached 0.8–1.0, at which point 0.1 mM isopropyl-β-D-1-thiogalactopyranoside (IPTG) was added, and expression was induced at 30 °C for 6 h. The recombinant chimeric proteins were purified using Ni-agarose resin (Sangon Biotech, Shanghai, China) via affinity chromatography, following procedures described in previous studies [39].

### 2.3. SDS-PAGE and Western Blot

Purified recombinant Fiber proteins were separated on a 12% Tris-Glycine PAGE gel. Proteins were transferred to a PVDF membrane, which was blocked with 5% bovine serum albumin (BSA; Sangon Biotech, Shanghai, China) for 2 h. The membrane was incubated with mouse anti-His monoclonal antibody (mAb) followed by horseradish peroxidase (HRP)-conjugated goat anti-mouse IgG (1:4000; TransGen, Beijing, China). The staining was carried out using DAB substrate solution, and the images were captured using an optical imaging system (Tanon3500BR, Shanghai, China). The concentration of the purified proteins was determined using a BCA Protein Assay Kit (Solarbio, Beijing, China).

### 2.4. Animal Experiment

Eighty-four 7-day-old SPF chickens were randomly assigned to seven groups (*n* = 12 per group): Fiber-C4/D11 vaccine group challenged with FAdV-4, Fiber-D11/C4 vaccine group challenged with FAdV-4, Fiber-C4/D11 vaccine group challenged with FAdV-11, Fiber-D11/C4 vaccine group challenged with FAdV-11, PBS/adjuvant group challenged with FAdV-4, PBS/adjuvant group challenged with FAdV-11, and the negative control group. The seven groups of SPF chickens were raised separately. Purified proteins were emulsified with 71VG adjuvant (SEPPIC, Montrouge, France) at a ratio of 3∶7 (*w*/*w*). Each milliliter of the vaccine contained 250 micrograms of the protein and was administered via subcutaneous injection in the neck at 0.1 mL on day 7, with a booster on day 14. Blood samples were collected on days 7, 14, 21, and 28 post vaccination (dpv) for IFA antibody and neutralizing antibody detection.

**Figure 1:**
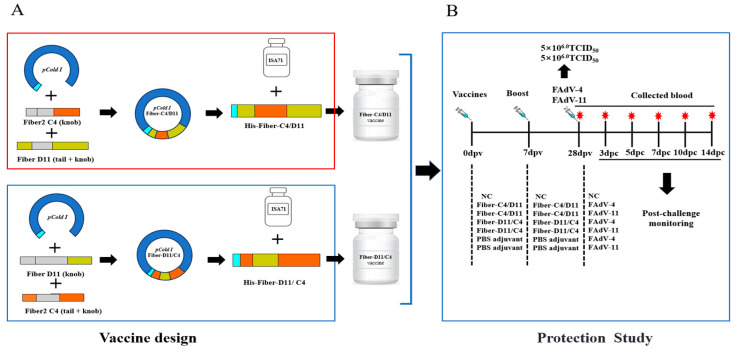
Design of subunit vaccines and experimental animal studies. (**A**) The construction and preparation of the chimeric vaccines. (**B**) The animal evaluation trials (days post-vaccination [dpv] and days post-challenge [dpc]), including initial vaccination, booster immunization, and challenge tests. Syringes indicate vaccination and challenge; red stars indicate blood collection from chickens.

At 28 dpv, the chickens were intramuscularly challenged with 0.5 mL of 10^6.0^ TCID_50_ of FAdV-4 or FAdV-11, while the negative control group received PBS. After the challenge, clinical symptoms, relative daily weight gain, and mortality were monitored for two weeks in all groups. At days 3, 5, 7, and 14 post-challenge (dpc), blood samples were collected for aspartate aminotransferase (AST) analysis. At these time points, three chickens from each group were also randomly selected for euthanasia and necropsy. Tissue organs and fecal swabs were collected for analysis. Organ-to-body weight ratios were calculated, and histopathological changes were assessed through stained tissue sections. The vaccine design and the protection study are shown in Figure 1.

### 2.5. Indirect Immunofluorescence Assay

LMH cells infected with FAdV-4 and FAdV-11 were fixed with a precooled acetone: ethanol (3:2, *v*/*v*) mixture at room temperature for 10 min. Cells were washed twice with PBS for 5 min each. Next, the cells were blocked with 1% BSA in PBST at 37 °C for 60 min, followed by two PBS washes for 5 min each. Immune chicken serum was added and incubated at 37 °C for 60 min. Afterward, the cells were washed four times, and FITC-conjugated goat anti-chicken was added and incubated in a 37 °C incubator for 60 min. The cells were washed four more times and observed under an inverted fluorescence microscope (Leica Microsystems, Wetzlar, Germany).

### 2.6. Enzyme-Linked Immunosorbent Assay and Serum Neutralization Test 

Serum samples were collected from the subunit vaccine groups at 7, 14, 21, and 28 dpv. The immunogenicity of the vaccine was assessed using the Enzyme-Linked Immunosorbent Assay (ELISA) and the neutralizing antibody titer determined with LMH cells. Plates were coated with Fiber-C4/D11 and Fiber-D11/C4 proteins at concentrations of 10 μg/mL and 12.5 μg/mL, respectively. The detection method was conducted as previously described [23]. The neutralization test was performed using a serum-fixed virus method. FAdV-4 strain SDSX and FAdV-11 strain SD, both at a concentration of 100 TCID_50_/0.1 mL, were mixed with an equal volume of two-fold serially diluted test sera. The mixtures were incubated and then inoculated onto well-grown LMH cells. The plates were incubated at 37 °C in 5% CO_2_ for 7 days. The titer of neutralizing antibodies was determined as the reciprocal of the highest serum dilution that completely inhibited the cytopathic effect (CPE).

### 2.7. Viral Titers in Target Tissues and Cloacal Swabs

To precisely quantify live viral loads in vivo, viral titers in cloacal swabs and tissue samples were measured using the TCID_50_ assay. Cloacal swabs were collected at 3, 5, 7, 10, and 14 dpc to assess viral shedding. Necropsy tissue samples were harvested at 3, 5, 7, and 14 dpc to determine viral loads in individual organs, facilitating a detailed evaluation of viral distribution and vaccine-mediated protection.

### 2.8. Statistical Analysis

Statistical analyses were performed using the GraphPad Prism 10 software (GraphPad Software, La Jolla, CA, USA). Group differences were assessed by two-way analysis of variance (Two-way ANOVA), followed by Tukey’s multiple comparisons test for post hoc analysis to adjust for the risk of type I error associated with multiple comparisons. Differences were considered statistically significant at *p* < 0.05.

## 3. Results

### 3.1. Construction, Expression and Purification of Recombinant Proteins

**Figure 2:**
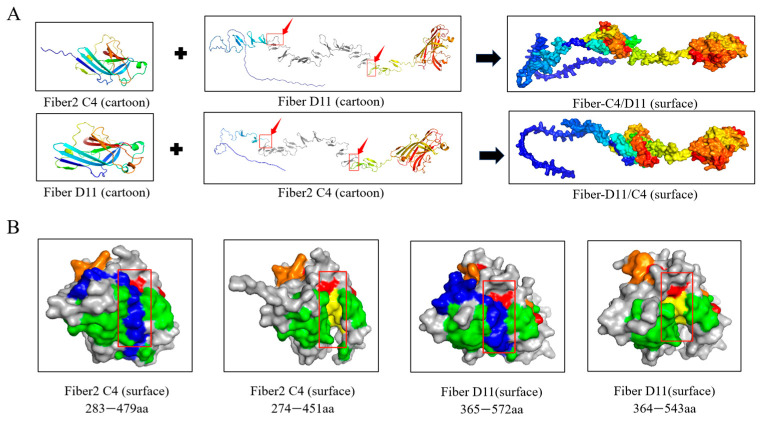
Strategy for the construction of recombinant proteins and three-dimensional model prediction of chimeric domains. The schematic illustrates the design principle for the chimeric proteins Fiber-C4/D11 and Fiber-D11/C4. Key neutralizing epitopes from different serotypes of Fiber proteins are swapped through domain swapping, and homology modeling is used to predict their spatial conformation. (**A**) The chimeric location of the Fiber protein and the surface images of the recombinant proteins Fiber-C4/D11 and Fiber-D11/C4 (red square indicated by the red arrow represents the chimeric exchange site). (**B**) Predicted structure and modeling of the knob domain amino acid sequence of Fiber protein (red area indicates the chimeric site; yellow and green areas represent previously reported epitopes [29,35]; yellow area indicates exposed neutralizing epitopes).

**Figure 3:**
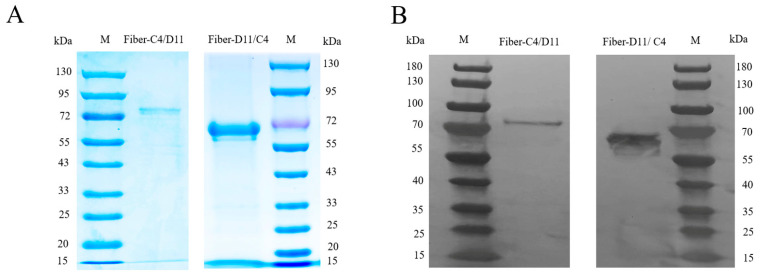
Expression and purification of recombinant proteins. (**A**) SDS-PAGE analysis of the purified recombinant proteins. M: protein marker. (**B**) Western blot analysis of the purified recombinant proteins (Fiber-C4/D11 and Fiber-D11/C4) probed by anti-His-monoclonal antibody. M: protein marker. (Original image is in Supplementary materials Figures S1 and S2).

Structural modeling revealed that the amino acid region 128–305 of the FAdV-11 Fiber protein (highlighted in a red frame) was replaced with the globular knob domain of FAdV-4 Fiber2 (amino acid residues 274–451). Conversely, the amino acid region 92–244 of the FAdV-4 Fiber protein (also highlighted in a red frame) was replaced with the knob domain of FAdV-11 Fiber (amino acid residues 364–543). The cartoon model of the chimeric proteins showed an overall conformation highly similar to that of the native Fiber protein, with no significant structural distortions or conformational changes, effectively preserving the integrity of the original three-dimensional structure and thereby maintaining the immunological activity of the knob domain (Figure 2A). In the chimeric design, the knob domain lacks the blue region amino acid sequence, leading to the exposure of neutralizing epitopes within the yellow regions—specifically amino acids 442–447 of FAdV-4 Fiber2 and amino acids 533–538 of FAdV-11 Fiber—which may be more readily recognized by the immune system. The green, yellow, and orange regions represent the epitope intervals previously reported in the literature [29,35], although the specific amino acid residues with neutralizing activity have not yet been definitively identified (Figure 2B). Subsequently, both chimeric proteins, Fiber-C4/D11 and Fiber-D11/C4, were successfully expressed in the *Escherichia coli* system and purified via affinity chromatography. SDS-PAGE and Western blot analyses showed that the purified proteins exhibited single, clear bands, indicating high purity, with concentrations of 1.0 mg/mL (Fiber-C4/D11) and 1.25 mg/mL (Fiber-D11/C4) (Figure 3A,B).

### 3.2. Indirect Immunofluorescence Assay (IFA)

**Figure 4:**
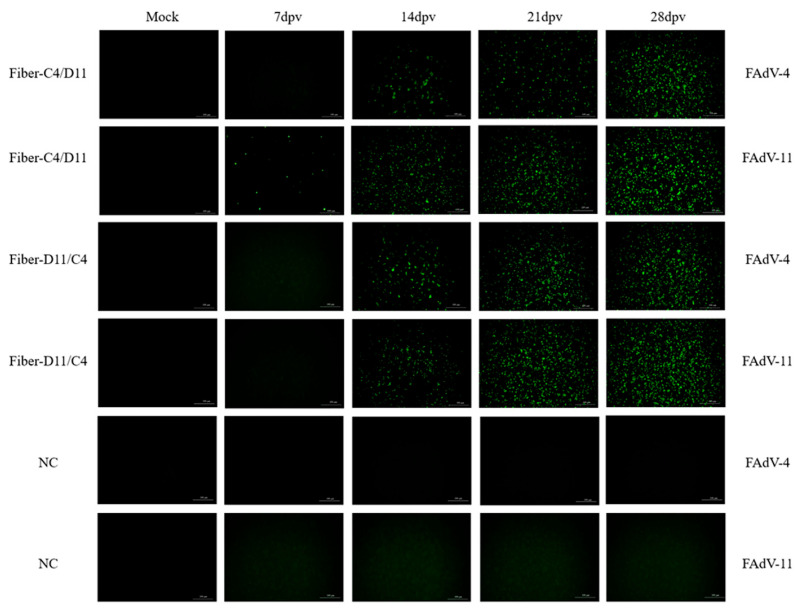
Antibodies were detected in chickens inoculated with the Fiber-C4/D11 and Fiber-D11/C4 subunit vaccines by IFA at 7, 14, 21, and 28 dpv.

Indirect immunofluorescence assay (IFA)was used to evaluate antibody levels in the serum samples from the four subunit vaccine groups at various time points post-immunization. Antibodies were detected in the Fiber-C4/D11 subunit vaccine group as early as 7 dpv when LMH cells were infected with FAdV-11 but not with FAdV-4. No antibodies were detected in the Fiber-D11/C4 groups at this time point. At 14, 21, and 28 dpv, antibodies were detected in both the Fiber-C4/D11 and Fiber-D11/C4 subunit vaccine groups when LMH cells were infected with FAdV-4 and FAdV-11 (Figure 4). These results indicate that the Fiber-C4/D11 subunit vaccine elicits an earlier antibody response compared to the Fiber-D11/C4 subunit vaccine.

### 3.3. Enzyme-Linked Immunosorbent Assay (ELISA) and Serum Neutralization Test (SNT)

**Figure 5:**
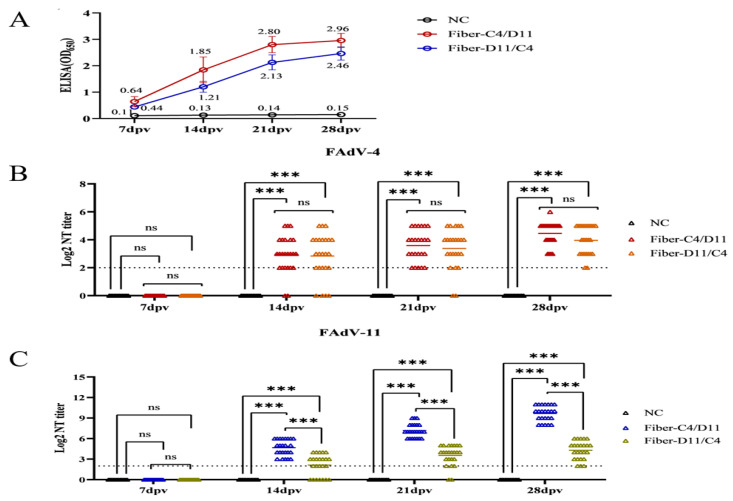
Serum antibody levels in chickens after immunization. (**A**) Antibody levels measured by ELISA at 7, 14, 21, and 28 dpv. (**B**) Serum neutralizing antibody titers against FAdV-4. (**C**) Serum neutralizing antibody titers against FAdV-11. Asterisks indicate significant differences between the Fiber-C4/D11, Fiber-D11/C4, and negative control (NC) groups (*** indicate *p* < 0.001).

**Figure 6:**
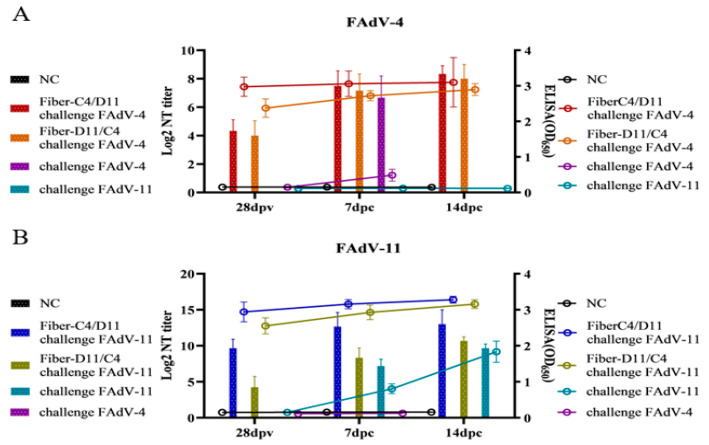
Serum antibody levels in chickens after challenge. (**A**) Antibody levels (ELISA and NTs) against FAdV-4. (**B**) Antibody levels (ELISA and NTs) against FAdV-11; in the ELISA tests, the Fiber-D11/C4 group was tested with the Fiber-D11/C4 protein, while all other groups were tested with the Fiber-C4/D11 protein.

The immunogenicity of the subunit vaccines was assessed using ELISA and serum neutralization tests (SNTs). The average ELISA optical density (OD) values for the Fiber-C4/D11 subunit vaccine were 0.64, 1.85, 2.80, and 2.96 at 7, 14, 21, and 28 dpv, respectively, compared to 0.44, 1.21, 2.13, and 2.46 for the Fiber-D11/C4 subunit vaccine. The negative control group exhibited consistently low OD values (<0.20) at all time points (Figure 5A). The Fiber-C4/D11 subunit vaccine induced significantly higher antibody levels than the Fiber-D11/C4 subunit vaccine at 14, 21, and 28 dpv (*p* < 0.05). Specific neutralizing antibodies were detected in both subunit vaccine groups at 14, 21, and 28 dpv. For FAdV-4, the Fiber-C4/D11 subunit vaccine group exhibited average neutralizing titers (NTs) of 2.95, 3.58, and 4.45 at 14, 21, and 28 dpv, respectively, compared to 2.83, 3.38, and 3.91 for the Fiber-D11/C4 subunit vaccine, with a significant difference at 28 dpv (Figure 5B, *p* < 0.05). For FAdV-11, the average NTs were 4.67, 7.16, and 9.66 for the Fiber-C4/D11 subunit vaccine and 2.08, 3.41, and 4.25 for the Fiber-D11/C4 subunit vaccine at 14, 21, and 28 dpv, respectively, with significantly higher titers in the Fiber-C4/D11 group (Figure 5C, *p* < 0.05, *p* < 0.01). Post-challenge, both subunit vaccine groups showed increased neutralizing antibody titers and ELISA OD values. Cross-neutralization tests confirmed the absence of cross-neutralizing antibodies, as the FAdV-4 challenge group did not induce neutralizing antibodies against FAdV-11, and vice versa (Figure 6A,B). These findings highlight the superior serotype-specific neutralizing antibody response elicited by the Fiber-C4/D11 subunit vaccine compared to the Fiber-D11/C4 subunit vaccine.

### 3.4. Clinical Protection

**Figure 7:**
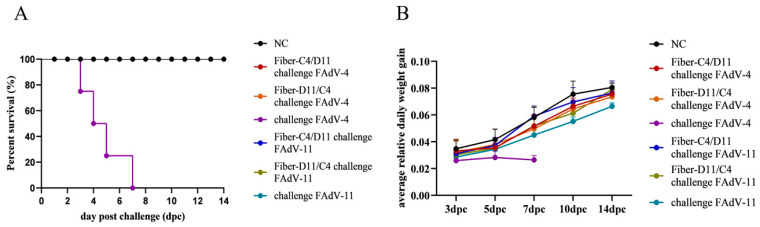
Survival curve and average daily relative weight gain of all chickens after FAdV challenge. (**A**) Survival curves of all chickens in challenge protection test following challenge with 5 × 10^6.0^ TCID_50_ FAdV-4 and 5 × 10^6.0^ TCID_50_ FAdV-11strain at 28 dpv. The survival rate of Fiber-C4/D11 challenge FAdV-4, Fiber-D11/C4 challenge FAdV-4, Fiber-C4/D11 challenge FAdV-11, Fiber-D11/C4 challenge FAdV-11, and challenge FAdV-11 was 100% and was covered by NC. (**B**) Average relative daily weight gain of all chickens during the challenge period.

Viral challenge experiments with FAdV-4 and FAdV-11 strains were conducted to evaluate the protective efficacy of the Fiber-C4/D11 and Fiber-D11/C4 subunit vaccines. No deaths or clinical symptoms were observed in the subunit vaccine or negative control groups. In contrast, the FAdV-4 challenge control group exhibited clinical symptoms starting at 2 dpc, including depression, anorexia, and prolonged recumbency, with mortality beginning on day 3, peaking on days 4 and 5, and reaching 100% by day 7. The FAdV-11 challenge control group showed no mortality but exhibited a slight decrease in appetite (Figure 7A). Weight gain analysis revealed significantly greater increases in the subunit vaccine and negative control groups compared to the FAdV-4 and FAdV-11 challenge control groups (Figure 7B, *p* < 0.05).

**Figure 8:**
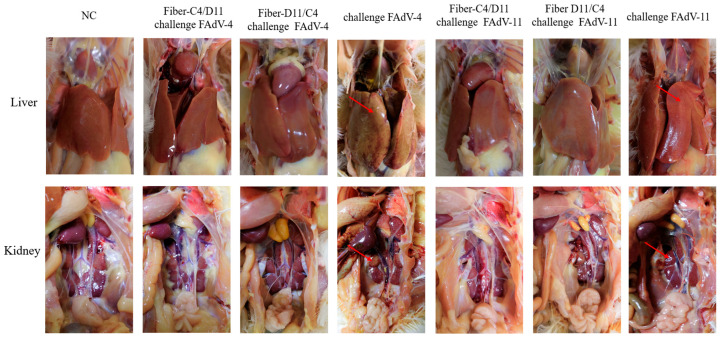
Pathological changes in SPF chickens after challenge. Livers in the FAdV-4 challenge control group were enlarged, yellowish, and brittle, with marginal hemorrhagic necrosis, and kidneys were enlarged and congested. In the FAdV-11 challenge control group, livers were enlarged and yellowish, with marginal hemorrhagic necrosis, and kidneys were congested. No clinical symptoms or pathological changes were observed in the negative control or subunit vaccine groups. Pathological changes in the liver and kidneys are indicated by red arrows.

Post-mortem examinations revealed no significant pathological changes in the subunit vaccine or negative control groups. In the FAdV-4 challenge control group, livers were enlarged, pale brown or yellowish, and friable and displayed hemorrhagic spots or patches, while the kidneys appeared enlarged and congested. In the FAdV-11 challenge control group, livers were enlarged, light yellow, and congested, with congested kidneys (Figure 8).

**Figure 9:**
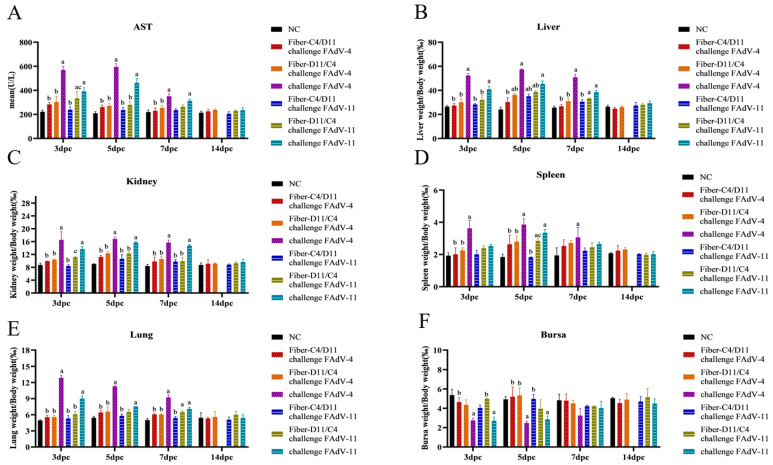
Plasma AST levels and organ-to-body weight ratios post-challenge. (**A**) Plasma AST levels for each experimental group. (**B**) Liver weight/body weight (LW/BW, ‰). (**C**) Kidney weight/body weight (KW/BW, ‰). (**D**) Spleen weight/body weight (SW/BW, ‰). (**E**) Lung weight/body weight (LW/BW, ‰). (**F**) Bursa weight/body weight (BW/BW, ‰). Lowercase letters above bars indicate significant differences (a, versus negative control; b, versus challenge FAdV-4 or FAdV-11 control; c, versus Fiber-C4/D11 challenge FAdV-4 or Fiber-C4/D11challenge FAdV-11). Data are presented as means ± standard deviation. The significance of differences was determined using two-way analysis of variance and significance was assessed at *p* < 0.05.

Aspartate aminotransferase (AST) levels were significantly higher in the Fiber-D11/C4 subunit vaccine group than in the Fiber-C4/D11 subunit vaccine group. Compared to the subunit vaccine and negative control groups, AST levels were markedly elevated in the FAdV-4 and FAdV-11 challenge control groups, peaking on day 5 (Figure 9A, *p* < 0.05). Organ-to-body weight ratios showed no significant differences in AST levels between the Fiber-C4/D11 and Fiber-D11/C4 subunit vaccine groups. However, at 3, 5, and 7 dpc, the relative weights of the liver, kidneys, spleen, and lungs were higher in the subunit vaccine and negative control groups, while the bursa weight-to-body ratio was significantly lower in the challenge control groups (Figure 9B–F, *p* < 0.05). These results suggest that the Fiber-C4/D11 subunit vaccine provides superior protection against FAdV-4 and FAdV-11 infections compared to the Fiber-D11/C4 subunit vaccine.

### 3.5. Histological Analysis

**Figure 10:**
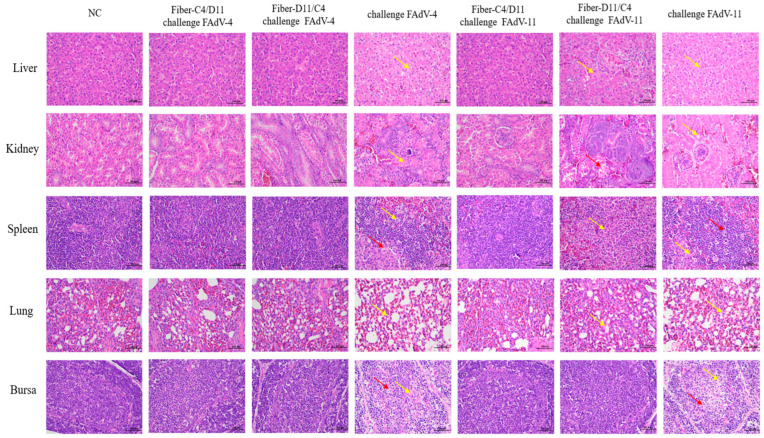
Histological analysis of liver, kidney, spleen, lung, and bursa tissues in chickens post-challenge (H&E stain, magnification ×400, scale bar = 100 μm). No histopathological alterations were observed in the negative control or Fiber-C4/D11 subunit vaccine groups. In the challenge control groups, widespread inflammatory cell infiltration, degeneration, and necrosis were observed (yellow arrows). Red blood cell congestion is indicated by red arrows.

Histopathological analysis revealed no significant pathological changes in the liver, kidney, spleen, bursa, or lung tissues of the vaccinated groups (Fiber-C4/D11 challenged with FAdV-4 or FAdV-11, and Fiber-D11/C4 challenged with FAdV-4) or the negative control group. In contrast, significant pathological alterations were observed in the Fiber-D11/C4 subunit vaccine group challenged with FAdV-11 and the two challenge control groups. In the challenge control groups, liver tissues exhibited lymphocyte infiltration, focal necrosis, and hepatocyte degeneration; renal tubular epithelial cells showed necrosis; and spleen and lung tissues displayed cellular swelling and inflammatory cell infiltration. Additionally, lymphocyte degeneration and extensive necrosis were observed in the cortical and medullary regions of the bursa. In the Fiber-D11/C4 subunit vaccine group challenged with FAdV-11, pathological changes were milder, primarily consisting of cellular swelling and lymphocyte infiltration in the kidneys, spleen, and bursa. These results indicate that the Fiber-C4/D11 subunit vaccine effectively protects against pathological changes caused by FAdV-4 and FAdV-11 infections, while the Fiber-D11/C4 subunit vaccine provides effective protection against FAdV-4 infection (Figure 10).

### 3.6. Viral Titers in Target Tissues and Cloacal Swabs

**Figure 11:**
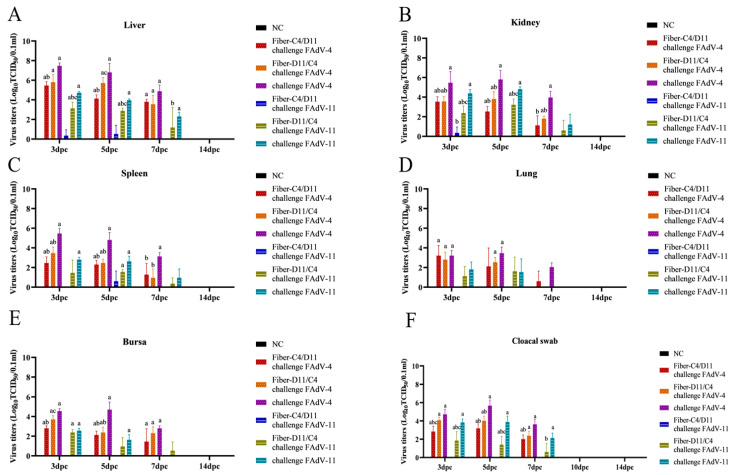
Viral titers in tissues and cloacal swabs post-challenge. (**A**) Liver. (**B**) Kidney (**C**) Spleen. (**D**) Lung. (**E**) Bursa. (**F**) Cloacal swabs. Viral titers were measured using TCID_50_ at 3, 5, 7, and 14 dpc. Lowercase letters above bars indicate significant differences (a, versus negative control; b, versus challenge FAdV-4 and FAdV-11 control; c, versus Fiber-C4/D11 group). Data are presented as means ± standard deviation. The significance of differences was determined using two-way analysis of variance and significance was assessed at *p* < 0.05.

Liver, kidney, spleen, bursa, lung tissues, and cloacal swabs were collected on days 3, 5, 7, and 14 dpc for viral titer determination using the TCID_50_ assay. At 3, 5, and 7 dpc, viral titers in the liver, kidney, spleen, and lung were significantly higher in the FAdV-4 and FAdV-11 challenge control groups compared to the subunit vaccine groups and negative control group. No viral titers were detected in the negative control group. The challenge control groups exhibited peak viral shedding at 3 and 5 dpc. Viral titers in the FAdV-4 challenge group were significantly higher than those in the FAdV-11 challenge group. Among the organs, the liver showed the highest viral shedding, followed by the kidney, spleen, lung, bursa, and cloacal swabs, with the lungs exhibiting the lowest viral titers. In the Fiber-C4/D11 vaccine group challenged with FAdV-11, virus was detected in the liver and kidneys of one bird at 3 dpc and in the liver and spleen of another bird at 5 dpc. However, no virus was detected in other organs or at later time points (7 dpc and 14 dpc). In contrast, in the Fiber-D11/C4 vaccine group, the virus was detected in multiple organs at both 3 dpc and 5 dpc. The Fiber-C4/D11 subunit vaccine group had significantly lower viral titers compared to the Fiber-D11/C4 subunit vaccine group (Figure 11A–F, *p* < 0.01). These results demonstrate that the Fiber-C4/D11 subunit vaccine is more effective at reducing viral titers in tissues and viral shedding compared to the Fiber-D11/C4 subunit vaccine.

## 4. Discussion

Outbreaks of fowl adenovirus (FAdV) have caused significant economic losses in the poultry industry, particularly affecting chicks aged 3 to 6 weeks. FAdV infections result in pathological changes, including pericardial effusion, enlarged and yellowed livers, tiger-striped kidneys, and enlarged spleens. Currently, inactivated vaccines are widely used to prevent hepatitis-hydropericardium syndrome (HHS) and inclusion body hepatitis (IBH) [16]. Among FAdV serotypes, FAdV-4 poses the greatest threat due to its high pathogenicity, and it has been the primary focus of vaccine development efforts [17]. However, the emergence of FAdV-2, -8a, -8b, and -11, combined with secondary and mixed infections, has driven increased research into multivalent vaccines. Although inactivated vaccines are rapidly produced, they have limitations, including incomplete inactivation, high production costs, and limited cross-protection capabilities, making it difficult to implement the “Differentiating Infected from Vaccinated Animals” (DIVA) strategy. Live vaccines, while capable of eliciting robust immune responses, pose risks of viral spread and can be horizontally transmitted to unvaccinated flocks, affecting biosafety control. In contrast, subunit vaccines offer superior safety and efficacy. The Fiber protein of FAdV is a key target for subunit vaccine development due to its ability to induce strong immune responses in chickens [31]. Notably, the epitope recognized by Mab3C2 on the Fiber-2 protein (amino acids 416–448) represents a conformational epitope with high neutralizing activity [29]. Schachner A et al.’s studies have also explored chimeric linear epitopes on the knob domain of FAdV-E, demonstrating protective effects against multiple viruses [35]. Variations in virulence among FAdV serotypes [40,41] have spurred interest in chimeric subunit vaccines, which offer protection against multiple serotypes [35].

Various strategies exist for constructing chimeric vaccines. For instance, De Luca et al. fused partial head domains of the FAdV-4 Fiber-2 protein with those of the FAdV-11 Fiber protein, resulting in a protein with some immune protection but no neutralizing antibody titers [24]. In contrast, our approach differs significantly. Using protein structure prediction and homology modeling, we identified that the Fiber-2 protein of FAdV-4 contains 13 β-turns in its tail and stem regions, while the FAdV-11 Fiber protein has 16 β-turns in similar regions. The increased number of β-turns and extended stem in FAdV-11 suggest greater structural capacity to incorporate immunogenic domains. This is supported by previous findings that the short and rigid Ad37 shaft would restrict knob binding to CAR at the cell surface, and that reducing β-turns impairs viral infectivity [42], whereas the globular knob domain of the Fiber protein elicits robust immune protection [37]. Leveraging these insights, we successfully constructed two chimeric Fiber proteins, Fiber-C4/D11 and Fiber-D11/C4, by exchanging their respective knob domains. These proteins achieved dual immunogenicity against FAdV-4 and FAdV-11 while avoiding significant conformational disruptions that could compromise immunogenicity. Both vaccines demonstrated strong immune responses.

At 7 dpv, indirect immunofluorescence assay (IFA) detected early antibodies against FAdV-11 in the Fiber-C4/D11 subunit vaccine group but not against FAdV-4. No antibodies were detected in the Fiber-D11/C4 group at this time point. First, immunological synergistic factors come into play: this may be attributed to the immunological properties of the Fiber-2 protein, as previous studies suggest that detectable antibodies require co-expression of Fiber-1 and Fiber-2 [36]. Second, structural factors have an impact: the shorter tail and stem domains of FAdV-4, with three fewer β-turns than FAdV-11, likely altered the protein’s spatial conformation, hindering the exposure of neutralizing epitopes in the Fiber-D11/C4 vaccine. Third, as regards immunological compensatory factors, current research indicates that cellular immunity is another crucial mechanism for effective protection against FAdV-related diseases such as HHS and IBH and can play a synergistic or compensatory role when humoral immunity is weak or delayed [24,34,40]. Therefore, despite the absence of early antibodies against FAdV-4, the Fiber-C4/D11 vaccine still provides effective protection, suggesting that the activation of cellular immunity may play an important protective role. Furthermore, regarding the potential adjuvant effect of the chimeric protein, there are few reports in the field of fowl adenovirus vaccines, and no clear literature supports the function of Fiber protein itself as a “self-adjuvant”. Nevertheless, we cannot rule out that the trimer structure or specific spatial arrangement of the chimeric protein in this study may have promoted antigen presentation and immune activation to some extent, a hypothesis that requires further verification in subsequent studies. At 14, 21, and 28 dpv, neutralizing antibodies (nAbs) against both FAdV-4 and FAdV-11 were detected in both vaccine groups. Notably, the Fiber-C4/D11 vaccine elicited significantly higher nAb titers against FAdV-11 compared to the Fiber-D11/C4 vaccine at all time points and against FAdV-4 at 28 dpv. The level of neutralizing antibodies induced by this vaccine differs from those reported previously. De Luca et al. found that subunit vaccines expressing only Fiber-2 could mainly detect neutralizing antibodies against FAdV-11, while the neutralizing response against FAdV-4 was almost undetectable [24]; the neutralizing antibody titer of this vaccine is also comparable to that of similar Fiber-2 subunit vaccines [34,36]. In contrast, multivalent live vaccines or inactivated vaccines (such as FAdV-4/8a/8b or FAdV-8a/8b/11) can induce higher neutralizing antibodies against different serotypes, but their cross-neutralization capabilities are generally weaker [21,31,43,44]. These results indicate that although the Fiber-C4/D11 vaccine is a single-antigen component, it shows potential for inducing cross-protection against different serotypes that is superior to that of traditional multivalent vaccines.

From the perspective of the incomplete head domain embedded in the knob domain, Wang P. et al.’s research has indicated that the amino acid region 416–448 of the FAdV-4 Fiber2 protein contains a key neutralizing epitope [29]. Notably, the epitopes formed by amino acids 442–447 of FAdV-4 Fiber2 and 533–538 of FAdV-11 Fiber in this study are precisely located within this functional region and are more fully exposed on the molecular surface in the chimeric protein. This structural feature may facilitate their effective recognition by the immune system, thereby inducing the production of neutralizing antibodies, suggesting that this region plays a significant role in mediating protective immune responses. Furthermore, De Luca et al.’s study found that when the epitope identified in this study, amino acids 442–447, was absent in the chimeric protein, neutralizing antibodies against FAdV-4 were virtually undetectable [24], further corroborating the critical role of this epitope in neutralization. In contrast, although the Fiber-D11/C4 vaccine retained the complete knob structure of FAdV-11 Fiber, it still induced lower levels of neutralizing antibodies, possibly due to spatial conformation restrictions or insufficient exposure of the key epitopes. The study indicates that the absence of amino acids 452–479 in the head domain of FAdV-4 Fiber2 and amino acids 544–572 in the head domain of FAdV-11 Fiber did not affect the production of cross-neutralizing antibodies. This finding is significant for the development of vaccines targeting multiple serotypes of FAdV, as it highlights the importance of epitope exposure and conformation in eliciting an effective immune response. Future research should focus on identifying the precise epitopes involved and understanding how their presentation can be optimized to enhance the generation of cross-neutralizing antibodies.

Challenge protection experiments corroborated these findings, showing a positive correlation between nAb titers and protective efficacy. Both vaccines protected chickens from mortality and significantly reduced viral loads in multiple tissues. Histopathological analyses further validated these outcomes (Figure 10), while significant differences in peak viral shedding at 3 and 5 dpc were observed between the vaccine and challenge control groups (Figure 11), with the liver’s role as a primary FAdV target aligning with prior research [44,45,46,47]. The Fiber-C4/D11 vaccine completely prevented FAdV-11 viral shedding, although neither vaccine fully blocked FAdV-4 shedding. Ongoing research is exploring whether increasing the concentration of the chimeric Fiber-C4/D11 protein could prevent FAdV-4 shedding. Antibody dynamics beyond 28 days post-immunization were not continuously monitored in this study. Whether the antibodies would significantly decline 6–8 weeks post-vaccination, as reported in the literature [35], remains to be further investigated. Inactivated vaccines based on FAdV-8a can provide partial cross-protection against FAdV-8b and FAdV-11 [21], suggesting that their Fiber proteins (especially the knob domain) may harbor conserved neutralizing epitopes. In the future, it may be possible to construct a multivalent chimeric vaccine covering major pathogenic serotypes such as FAdV-4, -11, -8a, and -8b, thereby more comprehensively addressing the complex and diverse infection pressures in the field.

Although the Fiber-C4/D11 chimeric vaccine has shown ideal immunogenicity and complete protective effects under experimental conditions, its cost control in large-scale production, process standardization, and actual effectiveness in complex field environments still need further verification, which will be the focus of the next phase of research and development. In addition, although cross-neutralizing reactions between different serotypes have been observed in inactivated vaccines, whether similar reactions can be induced after immunization with subunit Fiber protein vaccines, and their protective efficacy under real farming conditions, still requires in-depth exploration. In the future, the impact of optimizing the structural design of multivalent chimeric proteins or adjusting immunization dosages on the strength and duration of immune responses can be systematically evaluated to enhance the vaccine’s immunogenicity and long-term protective capacity. It is worth noting that in this study, no cross-neutralizing antibodies were detected between the FAdV-4 and FAdV-11 challenge groups, confirming the lack of natural cross-protection between these two serotypes, highlighting the necessity for developing broad-spectrum vaccines. However, both chimeric vaccines successfully induced cross-neutralizing reactions against heterologous serotypes, with the Fiber-C4/D11 vaccine showing superior immune protection. These results indicate that by rationally designing chimeric antigens, natural immune limitations can be overcome to actively induce cross-serotype protection. These findings emphasize the importance of chimeric subunit vaccines in the development of multivalent FAdV vaccines and offer a promising strategy to address the challenges posed by diverse FAdV serotypes.

## 5. Conclusions

In summary, based on protein structure prediction and homology modeling, we successfully constructed the chimeric Fiber-C4/D11 and Fiber-D11/C4 proteins. The chimeric Fiber-C4/D11 subunit vaccine elicited significantly higher levels of neutralizing antibody titers against FAdV-11 in SPF chickens and provided superior protection against both FAdV-4 and FAdV-11 infections in vivo compared to the Fiber-D11/C4 vaccine. This chimeric subunit vaccine achieved broad protection against both serotypes using a single antigen, marking a significant advancement in vaccine development. These findings offer a novel strategy for preventing and controlling fowl adenovirus infections, providing a promising solution to mitigate adenoviral diseases in the poultry industry.

## Data Availability

All available data are presented in this manuscript and Supplementary Materials.

## Supplementary Materials

The following supporting information can be downloaded at https://www.mdpi.com/article/10.3390/vetsci12090920/s1. Table S1: FAdV-4/11 gene cloning information list. Table S2: List of primers for cloning Fiber genes; the gene sequences and amino acid sequences of Fiber2 C4, Fiber D11, Fiber-C4/D11, and Fiber-D11/C4. Figure S1: Original Image of Figure 3A-Fiber-D11C4. Figure S2: Original Image of Figure 3A-Fiber-C4D11. Figure S3: Original Image of Figure 3B.
