# Supplementary material for: A Novel Chimeric Fiber-C4/D11 Subunit Vaccine Induces Cross-Neutralizing Antibodies and Provides Better Protection Against Fowl Adenovirus (FAdV) Type 4 and Type 11 Than the Fiber-D11/C4 Subunit Vaccine
Source: Vet Sci. 2025 Sep 22;12(9):920. doi: 10.3390/vetsci12090920 (PMC12474205; doi:10.3390/vetsci12090920)
Supplement: Supplementary file 1 [file vetsci-12-00920-s001.zip › Table S1 and S2.pdf]

Supplementary Explanation

Design of the recombinant chimera Fiber-C4/D11 and the recombinant complete Fiber-D11/C4. Overhangs at the 5′-termini of primer sequences with the vector sequence (Fiber-C4/D11 and Fiber-D11/C4 F/R) are represented by underlined nucleotides, and with the counterpart template sequence at the 3' end of the primer sequence.

Table S1. FAdV-4/11 gene cloning information list.

Designation of construct	Fragment (position in protein amino acid)	Reference Strain (GenBank accession number)	Primer Names	Expression vector (restriction sites used for cloning)
Fiber-C4/D11	Fiber D11 shaft (aa 1-127)	FAdV-4 reference strain (KY636400) FAdV-11 reference strain (PQ117790)	Fiber-C4/D11-F Fiber-C4/D11-R	pCold I (*HindIII*)
	Fiber-C4 knob (aa 274-451)			
	Fiber D11 knob (aa 306-572)			
Fiber-D11/C4	Fiber-C4 shaft (aa 1-91)		Fiber-D11/C4-F Fiber-D11/C4-R	
	Fiber D11 knob (aa 364-543)			
	Fiber-C4 knob (aa 245-479)			

Table S2. List of primers for cloning Fiber genes.

Primer Name	Sequences of Primers (5'-3')
Fiber D11/C4-F	CTC GAG GGA TCC GAA TTC AAG ATG GCG AAA TCG ACT CCT TTC
Fiber D11/C4-R	TCT AGA CTG CAG GTC GAC AAG GGG TTG TGT TAA TTT GTT GGT
Fiber-D11/C4-F	CTC GAG GGA TCC GAA TTC AAG ATG CTC CGG GCC CCT AAA AGA
Fiber-D11/C4-R	TCT AGA CTG CAG GTC GAC AAG CGG GAG GGA GGC CGC TGG ACA

Fowl aviadenovirus 4 isolate SDSX1, complete genome

GenBank: KY636400.1

FASTA Graphics

Go to:

LOCUS KY636400 43630 bp DNA circular VRL 17-JAN-2018

DEFINITION Fowl aviadenovirus 4 isolate SDSX1, complete genome.

ACCESSION KY636400

VERSION KY636400.1

KEYWORDS .
SOURCE Fowl aviadenovirus 4
ORGANISM Fowl aviadenovirus 4
Viruses; Varidnaviria; Bamfordvirae; Preplasmiviricota;
Tectiliviricetes; Rowavirales; Adenoviridae; Aviadenovirus; Fowl
aviadenovirus C.
REFERENCE 1 (bases 1 to 43630)
AUTHORS Chen,P., Yuan,W. and Sun,J.
TITLE Direct Submission
JOURNAL Submitted (17-FEB-2017) College of Veterinary Medicine,
Agricultural University of Hebei Province, Le Ken South Street,
Baoding 071000, China
COMMENT ##Assembly-Data-START##
Sequencing Technology :: Sanger dideoxy sequencing
##Assembly-Data-END##
FEATURES Location/Qualifiers
source 1..43630
/organism="Fowl aviadenovirus 4"
/mol_type="genomic DNA"
/isolate="SDSX1"
/host="fowl"
/db_xref="taxon:130663"
/country="China"
/collection_date="26-Sep-2015"
/collected_by="Yuan Wanzhe"
CDS 31726..33165
/codon_start=1
/product="**Fiber-2 protein**"
/protein_id="AUO29792.1"
/translation="MLRAPKRRHSENGKPETEAGPSPAPIKRAKRMVRASQLDLVYPF
DYVADPVGGLNPPFLGGSGPLVDQGGQLTLNVTDPIIIKNRSVDLAHDPSLDVNAQGQ
LAVAVDPEGALDITPDGLDVKVDGVTVMVNDDWELAVKVDPSGGLDSTAGGLGVSVDD
TLLVDQGELGVHLNQQGPITADSSGIDLEINPNMFTVNTSTGSGVLELNLKAQGGIQA
DSSGVGVSVDESLQIVNNTLEVKPDPSGPLTVSANGLGLKYDTNTLAVTAGALTVVGG
GSVSTPIATFVSGSPSLNTYNATTVNSSANAFSCAYYLQQWNIQGLLVTSLYLKLDSA
TMGNRPGDLNSANAKWFTFWVSAYLQQCNPSGIQAGTVSPSTATLTDFEPMANRSVTS
PWTYSANGYYEPSIGEFQVFSPVVTGAWNPGNIGIRVLPVPVSASGERYTLLCYSLQC
TNASIFNPNNSGTMIVGPVLYSCPAASLP"

sequence of Fiber2 knob

ATGCTCCGGGCCCCTAAAAGAAGACATTCCGAAAACGGGAAGCCCGAGACCGAAGCGGGACCTTCCC
CGGCTCCAATCAAGCGCGCCAAACGCATGGTGAGAGCATCCCAGCTTGACCTGGTTTATCCTTTCGATT
ACGTGGCCGACCCCGTCGGAGGGCTCAACCCGCCTTTTTTGGGAGGCTCAGGACCCCTAGTGGACCA
GGGCGGACAGCTTACGCTCAACGTCACCGATCCCATCATCATCAAGAACAGATCGGTGGACTTGGCCC

ACGACCCCAGTCTCGATGTCAACGCCCAAGGTCAACTGGCGGTGGCCGTTGACCCCGAAGGGGCCCT
GGACATCACCCCCGATGGACTGGACGTCAAGGTCGACGGAGTGACCGTAATGGTCAACGATGACTGG
GAACTGGCCGTAAAAGTCGACCCGTCCGGCGGATTGGATTCCACCGCGGGTGGACTGGGGGTCAGCG
TGGACGACACCTTGCTCGTGGATCAGGGAGAACTGGGCGTACACCTCAACCAACAAGGACCCATCAC
TGCCGATAGCAGTGGTATCGACCTCGAGATCAATCCTAACATGTTCACGGTCAACACCTCGACCGGAA
GCGGAGTGCTGGAACTCAACCTAAAAGCGCAGGGAGGCATCCAAGCCGACAGTTCGGGAGTGGGCG
TTTCCGTGGATGAAAGCCTACAGATTGTCAACAACACTCTGGAAGTGAAACCGGATCCCAGCGGACC
GCTTACGGTCTCCGCCAATGGCCTAGGGCTGAAGTACGACACTAATACCCTAGCGGTGACCGCGGGCG
CTTTAACCGTGGTCGGAGGGGGGAGCGTCTCCACACCCATCGCTACTTTTGTCTCGGGAAGTCCCAGC
CTCAACACCTACAATGCCACGACCGTCAATTCCAGCGCGAACGCCTTCTCTTGCGCCTACTACCTTCA
ACAGTGGAACATACAGGGGCTCCTTGTTACCTCCCTCTACTTGAAATTGGACAGCGCCACCATGGGGA
ATCGCCCTGGGGACCTCAACTCCGCCAATGCCAAATGGTTCACCTTTTGGGTGTCCGCCTATCTCCAGC
AATGCAACCCCTCCGGGATTCAAGCGGGAACGGTCAGCCCCTCCACCGCCACCCTCACGGACTTTGA
ACCCATGGCCAATAGGAGCGTGACCAGCCCATGGACGTACTCGGCCAATGGATACTATGAACCATCCAT
CGGGGAATTCCAAGTGTTCAGCCCGGTGGTAACAGGTGCCTGGAACCCGGGAAACATAGGGATCCGC
GTCCTCCCCGTGCCGGTTTCGGCCTCCGGAGAGCGATACACCCTTCTATGCTATAGTCTGCAGTGCACG
AACGCGAGCATTTTTAATCCAAACAACAGCGGAACCATGATCGTGGGACCCGTGCTCTACAGCTGTCC
AGCGGCCTCCCTCCCG

Fowl aviadenovirus D isolate SD/2021, complete genome

GenBank: PQ117790.1

FASTA Graphics PopSet

LOCUS PQ117790 44362 bp DNA linear VRL 09-DEC-2024

DEFINITION Fowl aviadenovirus D isolate SD/2021, complete genome.

ACCESSION PQ117790

VERSION PQ117790.1

KEYWORDS .

SOURCE Fowl aviadenovirus D

ORGANISM Fowl aviadenovirus D

Viruses; Varidnaviria; Bamfordvirae; Preplasmiviricota;
Tectiliviricetes; Rowavirales; Adenoviridae; Aviadenovirus.

REFERENCE 1 (bases 1 to 44362)

AUTHORS Wang,X.Q.

TITLE Direct Submission

JOURNAL Submitted (30-JUL-2024) Research and Development Center, Shandong
Sinder Technology Co., LTD, No. 335 Songling Road, Laoshan
District, Qingdao, Shandong 266100, China

COMMENT ##Assembly-Data-START##
Sequencing Technology :: Sanger dideoxy sequencing
##Assembly-Data-END##

FEATURES Location/Qualifiers

source 1..44362

/organism="Fowl aviadenovirus D"

/mol_type="genomic DNA"
/isolate="SD/2021"
/host="chicken"
/db_xref="taxon:190064"
/geo_loc_name="China"
/collection_date="04-Jun-2021"

CDS 30186..31904
/codon_start=1
/product="fiber protein"
/protein_id="XHO48104.2"

/translation="MAKSTPFTFSMGQHSSRKRPADSENTQNASKVAKTQTSATRAGVDGNDDLNLV
YPFWLQNSTSGGGGGGSGGNPSLNPPFIDPNGPLYVQNSLLYVKTTAPIEVENKSLALAYDSSL
DVDAQNQLQVKVDAEGPIRISPDGLDIAVDPSTLEVDDEWELTVKLDPAGPISSSSAGINIRVD
DTLLIEDDDTAQVKELGVHLNPNGPITADQDGLDLEVDPQTLTVTTSGATGGVLGVLLKPSGG
LQTSIQGIGVAVADTLTISSNTGTVEVKTDPNGSIGSSSNGIAVVTDPAGPLTTSSNGLSLKLTPN
GSIQSSSTGLSVQTDPAGPITSGANGLSLSYDTSDFTVSQGMLSIIRNPSAYPDAYLESGTNLLN
NYTAYAENSSNYKFNCAYFLQSWYSNGLVTSSLYLKINRDNLTSLPSGQLSENAKYFTFWVPT
YESMNLSNVATPTITPSSVPWGAFLPAQNCTSNPAFKYYLTQPPSIYFEPESGSVQTFQPVLTGD
WDTNTYNPGTVQVCILPQTVVGGQSTFVNMTCYNFRCQNPGIFKVAASSGTFTIGPIFYSCPTN
KLTQP"

Optimized sequence of Fiber D11

ATGGCGAAATCGACTCCTTTCACGTTCTCCATGGGACAGCACTCCAGCCGAAAACGTCCC
GCGGACAGCGAAAACACGCAAAATGCATCAAAAGTCGCCAAAACGCAGACTTCTGCCAC
GCGCGCCGGTGTCGACGGAAATGACGACCTAAACCTGGTGTACCCCTTTTGGCTCCAAAA
CAGCACTTCGGGGGGCGGAGGAGGAGGAAGCGGCGGAAACCCCTCCCTAAATCCCCCTTT
TATCGACCCTAACGGACCCCTCTATGTTCAAAACAGTCTCCTTTATGTCAAAACTACTGCA
CCTATCGAGGTTGAAAACAAGTCACTCGCCCTAGCTTATGATTCCTCACTGGATGTGGACG
CTCAAAATCAGCTACAAGTGAAGGTAGATGCCGAGGGACCCATCAGGATTTCCCCAGATG
GGCTCGATATTGCTGTCGACCCATCGACGTTGGAGGTTGATGATGAATGGGAGCTGACCG
TCAAACTCGACCCGGCCGGGCCCATATCCTCATCCTCTGCCGGAATCAACATACGAGTAG
ATGATACGCTCTTAATCGAAGACGATGACACCGCCCAAGTTAAAGAATTAGGCGTGCATC
TCAACCCCAACGGCCCCATTACCGCTGACCAAGATGGGTTGGACTTAGAAGTGGACCCAC
AAACTTTAACCGTCACTACCAGCGGAGCCACGGGAGGAGTTCTAGGAGTACTTCTGAAAC
CCAGCGGGGGGTTACAGACAAGCATTCAGGGTATCGGAGTCGCCGTAGCAGATACTTTAA
CTATATCGAGTAACACGGGGACGGTCGAAGTAAAAACCGATCCCAACGGTTCTATTGGAT
CCTCTAGCAATGGGATAGCAGTAGTTACCGATCCCGCAGGTCCGCTCACTACTTCATCTAA
CGGTCTGTCTCTGAAATTAACACCGAACGGTTCCATCCAATCATCAAGTACGGGCCTATCC
GTCCAGACCGATCCCGCGGGACCCATTACCTCCGGTGCCAACGGACTAAGCTTATCCTAC
GACACTTCTGACTTTACAGTAAGTCAAGGAATGCTCAGTATCATACGGAATCCAAGCGCC
TATCCAGATGCTTATCTAGAATCGGGAACCAACTTACTGAATAATTACACGGCTTATGCCG
AAAACTCCAGTAATTACAAGTTTAACTGCGCTTATTTTCTTCAGTCCTGGTATTCCAACGG
ACTAGTGACTTCCTCCCTTTATCTCAAAATCAACAGGGATAACCTCACTAGTCTACCTTCT

GGTCAATTAAGTGAAAATGCCAAATATTTTACATTTTGGGTGCCCACCTATGAATCGATGA
ACCTTTCCAATGTTGCAACACCTACTATTACCCCTAGCAGCGTCCCGTGGGGAGCATTCTT
ACCCGCACAAAATTGCACGAGTAATCCCGCCTTTAAGTACTATCTCACGCAACCGCCAAG
CATCTATTTCGAGCCAGAATCGGGTTCCGTGCAAACTTTCCAACCCGTATTGACAGGAGAT
TGGGATACCAACACCTACAACCCAGGAACCGTTCAAGTCTGCATACTGCCTCAAACCGTT
GTGGGAGGCCAGTCGACCTTTGTTAACATGACATGTTATAACTTCCGGTGTCAAAATCCTG
GAATATTCAAGGTTGCTGCTAGTAGCGGCACATTCACTATCGGACCCATTTTCTACTCCTG
TCCAACCAACAAATTAACACAACCC

Sequence of Fiber2 C4 knob

GTCGGAGGGGGGAGCGTCTCCACACCCATCGCTACTTTTGTCTCGGGAAGTCCCAGCC
TCAACACCTACAATGCCACGACCGTCAATTCCAGCGCGAACGCCTTCTCTTGCGCCTA
CTACCTTCAACAGTGGAACATACAGGGGCTCCTTGTTACCTCCCTCTACTTGAAATTGG
ACAGCGCCACCATGGGGAATCGCCCTGGGGACCTCAACTCCGCCAATGCCAAATGGTT
CACCTTTTGGGTGTCCGCCTATCTCCAGCAATGCAACCCCTCCGGGATTCAAGCGGGA
ACGGTCAGCCCCTCCACCGCCACCCTCACGGACTTTGAACCCATGGCCAATAGGAGCG
TGACCAGCCCATGGACGTACTCGGCCAATGGATACTATGAACCATCCATCGGGGAATTC
CAAGTGTTCAGCCCGGTGGTAACAGGTGCCTGGAACCCGGGAAACATAGGGATCCGC
GTCCTCCCCGTGCCGGTTTCGGCCTCCGGAGAGCGATACACCCTTCTATGCTATAGTCT
GCAGTGCACG

Sequence of Fiber D11 (shaft + knob)

ATGGCGAAATCGACTCCTTTCACGTTCTCCATGGGACAGCACTCCAGCCGAAAACGTC
CCGCGGACAGCGAAAACACGCAAAATGCATCAAAAGTCGCCAAAACGCAGACTTCTG
CCACGCGCGCCGGTGTCGACGGAAATGACGACCTAAACCTGGTGTACCCCTTTTGGCT
CCAAAACAGCACTTCGGGGGGCGGAGGAGGAGGAAGCGGCGGAAACCCCTCCCTAA
ATCCCCCTTTTATCGACCCTAACGGACCCCTCTATGTTCAAAACAGTCTCCTTTATGTCA
AAACTACTGCACCTATCGAGGTTGAAAACAAGTCACTCGCCCTAGCTTATGATTCCTCA
CTGGATGTGGACGCTCAAAATCAGCTACAAGTGAAATTAACACCGAACGGTTCCATCC
AATCATCAAGTACGGGCCTATCCGTCCAGACCGATCCCGCGGGACCCATTACCTCCGGT
GCCAACGGACTAAGCTTATCCTACGACACTTCTGACTTTACAGTAAGTCAAGGAATGCT
CAGTATCATACGGAATCCAAGCGCCTATCCAGATGCTTATCTAGAATCGGGAACCAACT
TACTGAATAATTACACGGCTTATGCCGAAAACTCCAGTAATTACAAGTTTAACTGCGCT
TATTTTCTTCAGTCCTGGTATTCCAACGGACTAGTGACTTCCTCCCTTTATCTCAAAATC
AACAGGGATAACCTCACTAGTCTACCTTCTGGTCAATTAAGTGAAAATGCCAAATATTT
TACATTTTGGGTGCCCACCTATGAATCGATGAACCTTTCCAATGTTGCAACACCTACTAT
TACCCCTAGCAGCGTCCCGTGGGGAGCATTCTTACCCGCACAAAATTGCACGAGTAAT
CCCGCCTTTAAGTACTATCTCACGCAACCGCCAAGCATCTATTTCGAGCCAGAATCGGG
TTCCGTGCAAACTTTCCAACCCGTATTGACAGGAGATTGGGATACCAACACCTACAAC
CCAGGAACCGTTCAAGTCTGCATACTGCCTCAAACCGTTGTGGGAGGCCAGTCGACCT
TTGTTAACATGA
CATGTTATAACTTCCGGTGTCAAAATCCTGGAATATTCAAGGTTGCTGCTAGTAGCGGC
ACATTCACTATCGGACCCATTTTCTACTCCTGTCCAACCAACAAATTAACACAACCC

Sequence of Fiber C4/D11

ATGGCGAAATCGACTCCTTTCACGTTCTCCATGGGACAGCACTCCAGCCGAAAACGTC
CCGCGGACAGCGAAAACACGCAAAATGCATCAAAAGTCGCCAAAACGCAGACTTCTG
CCACGCGCGCCGGTGTCGACGGAAATGACGACCTAAACCTGGTGTACCCCTTTTGGCT
CCAAAACAGCACTTCGGGGGGCGGAGGAGGAGGAAGCGGCGGAAACCCCTCCCTAA
ATCCCCCTTTTATCGACCCTAACGGACCCCTCTATGTTCAAAACAGTCTCCTTTATGTCA
AAACTACTGCACCTATCGAGGTTGAAAACAAGTCACTCGCCCTAGCTTATGATTCCTCA
CTGGATGTGGACGCTCAAAATCAGCTACAAGTGGTCGGAGGGGGGAGCGTCTCCACA
CCCATCGCTACTTTTGTCTCGGGAAGTCCCAGCCTCAACACCTACAATGCCACGACCGT
CAATTCCAGCGCGAACGCCTTCTCTTGCGCCTACTACCTTCAACAGTGGAACATACAG
GGGCTCCTTGTTACCTCCCTCTACTTGAAATTGGACAGCGCCACCATGGGGAATCGCCC
TGGGGACCTCAACTCCGCCAATGCCAAATGGTTCACCTTTTGGGTGTCCGCCTATCTCC
AGCAATGCAACCCCTCCGGGATTCAAGCGGGAACGGTCAGCCCCTCCACCGCCACCCT
CACGGACTTTGAACCCATGGCCAATAGGAGCGTGACCAGCCCATGGACGTACTCGGCC
AATGGATACTATGAACCATCCATCGGGGAATTCCAAGTGTTCAGCCCGGTGGTAACAGG
TGCCTGGAACCCGGGAAACATAGGGATCCGCGTCCTCCCCGTGCCGGTTTCGGCCTCC
GGAGAGCGATACACCCTTCTATGCTATAGTCTGCAGTGCACGAAATTAACACCGAACG
GTTCCATCCAATCATCAAGTACGGGCCTATCCGTCCAGACCGATCCCGCGGGACCCATT
ACCTCCGGTGCCAACGGACTAAGCTTATCCTACGACACTTCTGACTTTACAGTAAGTCA
AGGAATGCTCAGTATCATACGGAATCCAAGCGCCTATCCAGATGCTTATCTAGAATCGG
GAACCAACTTACTGAATAATTACACGGCTTATGCCGAAAACTCCAGTAATTACAAGTTT
AACTGCGCTTATTTTCTTCAGTCCTGGTATTCCAACGGACTAGTGACTTCCTCCCTTTAT
CTCAAAATCAACAGGGATAACCTCACTAGTCTACCTTCTGGTCAATTAAGTGAAAATGC
CAAATATTTTACATTTTGGGTGCCCACCTATGAATCGATGAACCTTTCCAATGTTGCAAC
ACCTACTATTACCCCTAGCAGCGTCCCGTGGGGAGCATTCTTACCCGCACAAAATTGCA
CGAGTAATCCCGCCTTTAAGTACTATCTCACGCAACCGCCAAGCATCTATTTCGAGCCA
GAATCGGGTTCCGTGCAAACTTTCCAACCCGTATTGACAGGAGATTGGGATACCAACA
CCTACAACCCAGGAACCGTTCAAGTCTGCATACTGCCTCAAACCGTTGTGGGAGGCCA
GTCGACCTTTGTTAACATGACATGTTATAACTTCCGGTGTCAAAATCCTGGAATATTCAA
GGTTGCTGCTAGTAGCGGCACATTCACTATCGGACCCATTTTCTACTCCTGTCCAACCA
ACAAATTAACACAACCC

Sequence of Fiber D11 knob

GATGCTTATCTAGAATCGGGAACCAACTTACTGAATAATTACACGGCTTATGCCGAAAA
CTCCAGTAATTACAAGTTTAACTGCGCTTATTTTCTTCAGTCCTGGTATTCCAACGGACT
AGTGACTTCCTCCCTTTATCTCAAAATCAACAGGGATAACCTCACTAGTCTACCTTCTG
GTCAATTAAGTGAAAATGCCAAATATTTTACATTTTGGGTGCCCACCTATGAATCGATGA
ACCTTTCCAATGTTGCAACACCTACTATTACCCCTAGCAGCGTCCCGTGGGGAGCATTC
TTACCCGCACAAAATTGCACGAGTAATCCCGCCTTTAAGTACTATCTCACGCAACCGCC
AAGCATCTATTTCGAGCCAGAATCGGGTTCCGTGCAAACTTTCCAACCCGTATTGACAG
GAGATTGGGATACCAACACCTACAACCCAGGAACCGTTCAAGTCTGCATACTGCCTCA

AACCGTTGTGGGAGGCCAGTCGACCTTTGTTAACATGACATGTTATAACTTCCGGTGTC
AAAATCCT

Sequence of Fiber2 C4 (shaft + knob)

ATGCTCCGGGCCCCTAAAAGAAGACATTCCGAAAACGGGAAGCCCGAGACCGAAGCG
GGACCTTCCCCGGCTCCAATCAAGCGCGCCAAACGCATGGTGAGAGCATCCCAGCTTG
ACCTGGTTTATCCTTTCGATTACGTGGCCGACCCCGTCGGAGGGCTCAACCCGCCTTTT
TTGGGAGGCTCAGGACCCCTAGTGGACCAGGGCGGACAGCTTACGCTCAACGTCACC
GATCCCATCATCATCAAGAACAGATCGGTGGACTTGGCCCACTAAAAGTCGACCCGTC
CGGCGGATTGGATTCCACCGCGGGTGGACTGGGGGTCAGCGTGGACGACACCTTGCT
CGTGGATCAGGGAGAACTGGGCGTACACCTCAACCAACAAGGACCCATCACTGCCGA
TAGCAGTGGTATCGACCTCGAGATCAATCCTAACATGTTCACGGTCAACACCTCGACCG
GAAGCGGAGTGCTGGAACTCAACCTAAAAGCGCAGGGAGGCATCCAAGCCGACAGTT
CGGGAGTGGGCGTTTCCGTGGATGAAAGCCTACAGATTGTCAACAACACTCTGGAAGT
GAAACCGGATCCCAGCGGACCGCTTACGGTCTCCGCCAATGGCCTAGGGCTGAAGTAC
GACACTAATACCCTAGCGGTGACCGCGGGCGCTTTAACCGTGGTCGGAGGGGGGAGC
GTCTCCACACCCATCGCTACTTTTGTCTCGGGAAGTCCCAGCCTCAACACCTACAATGC
CACGACCGTCAATTCCAGCGCGAACGCCTTCTCTTGCGCCTACTACCTTCAACAGTGG
AACATACAGGGGCTCCTTGTTACCTCCCTCTACTTGAAATTGGACAGCGCCACCATGGG
GAATCGCCCTGGGGACCTCAACTCCGCCAATGCCAAATGGTTCACCTTTTGGGTGTCC
GCCTATCTCCAGCAATGCAACCCCTCCGGGATTCAAGCGGGAACGGTCAGCC

Sequence of Fiber-D11/C4

ATGCTCCGGGCCCCTAAAAGAAGACATTCCGAAAACGGGAAGCCCGAGACCGAAGCG
GGACCTTCCCCGGCTCCAATCAAGCGCGCCAAACGCATGGTGAGAGCATCCCAGCTTG
ACCTGGTTTATCCTTTCGATTACGTGGCCGACCCCGTCGGAGGGCTCAACCCGCCTTTT
TTGGGAGGCTCAGGACCCCTAGTGGACCAGGGCGGACAGCTTACGCTCAACGTCACC
GATCCCATCATCATCAAGAACAGATCGGTGGACTTGGCCCACGATGCTTATCTAGAATC
GGGAACCAACTTACTGAATAATTACACGGCTTATGCCGAAAACTCCAGTAATTACAAGT
TTAACTGCGCTTATTTTCTTCAGTCCTGGTATTCCAACGGACTAGTGACTTCCTCCCTTT
ATCTCAAAATCAACAGGGATAACCTCACTAGTCTACCTTCTGGTCAATTAAGTGAAAAT
GCCAAATATTTTACATTTTGGGTGCCCACCTATGAATCGATGAACCTTTCCAATGTTGCA
ACACCTACTATTACCCCTAGCAGCGTCCCGTGGGGAGCATTCTTACCCGCACAAAATTG
CACGAGTAATCCCGCCTTTAAGTACTATCTCACGCAACCGCCAAGCATCTATTTCGAGC
CAGAATCGGGTTCCGTGCAAACTTTCCAACCCGTATTGACAGGAGATTGGGATACCAA
CACCTACAACCCAGGAACCGTTCAAGTCTGCATACTGCCTCAAACCGTTGTGGGAGGC
CAGTCGACCTTTGTTAACATGACATGTTATAACTTCCGGTGTCAAAATCCTTAAAAGTC
GACCCGTCCGGCGGATTGGATTCCACCGCGGGTGGACTGGGGGTCAGCGTGGACGAC
ACCTTGCTCGTGGATCAGGGAGAACTGGGCGTACACCTCAACCAACAAGGACCCATC
ACTGCCGATAGCAGTGGTATCGACCTCGAGATCAATCCTAACATGTTCACGGTCAACAC
CTCGACCGGAAGCGGAGTGCTGGAACTCAACCTAAAAGCGCAGGGAGGCATCCAAGC
CGACAGTTCGGGAGTGGGCGTTTCCGTGGATGAAAGCCTACAGATTGTCAACAACACT
CTGGAAGTGAAACCGGATCCCAGCGGACCGCTTACGGTCTCCGCCAATGGCCTAGGGC
TGAAGTACGACACTAATACCCTAGCGGTGACCGCGGGCGCTTTAACCGTGGTCGGAGG

GGGGAGCGTCTCCACACCCATCGCTACTTTTGTCTCGGGAAGTCCCAGCCTCAACACC
TACAATGCCACGACCGTCAATTCCAGCGCGAACGCCTTCTCTTGCGCCTACTACCTTCA
ACAGTGGAACATACAGGGGCTCCTTGTTACCTCCCTCTACTTGAAATTGGACAGCGCC
ACCATGGGGAATCGCCCTGGGGACCTCAACTCCGCCAATGCCAAATGGTTCACCTTTT
GGGTGTCCGCCTATCTCCAGCAATGCAACCCCTCCGGGATTCAAGCGGGAACGGTCAG
CC

Note: Yellow letters are the inserted fragment.

Note: The insertion site is between two green letters.
