# Supplementary figures and images for: A Novel Chimeric Fiber-C4/D11 Subunit Vaccine Induces Cross-Neutralizing Antibodies and Provides Better Protection Against Fowl Adenovirus (FAdV) Type 4 and Type 11 Than the Fiber-D11/C4 Subunit Vaccine
Source: Vet Sci. 2025 Sep 22;12(9):920. doi: 10.3390/vetsci12090920 (PMC12474205; doi:10.3390/vetsci12090920)

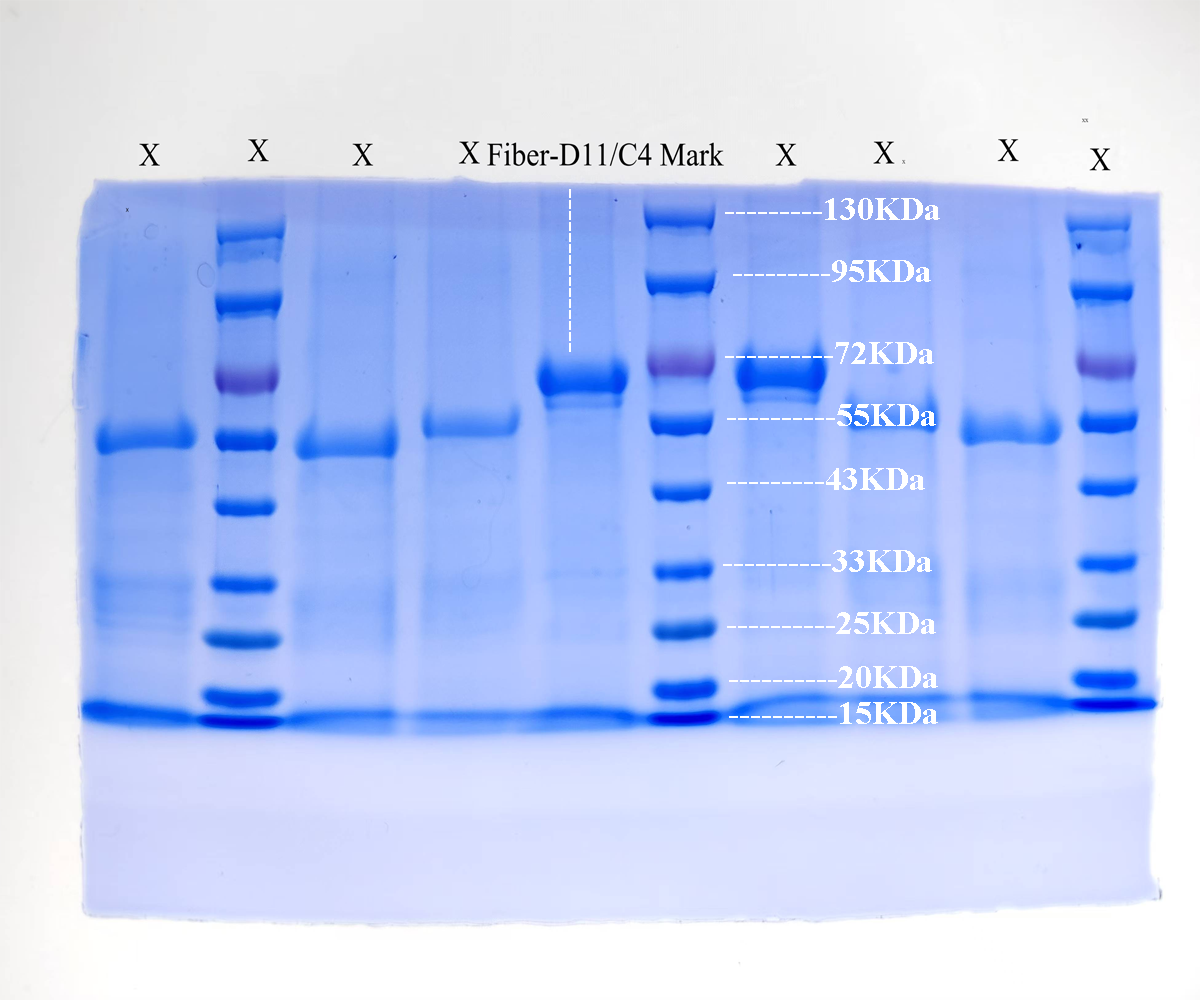

Supplement: Supplementary file 1 [file vetsci-12-00920-s001.zip › Figure S1 - Original Figure 3 -A--Fiber-D11C4.png]

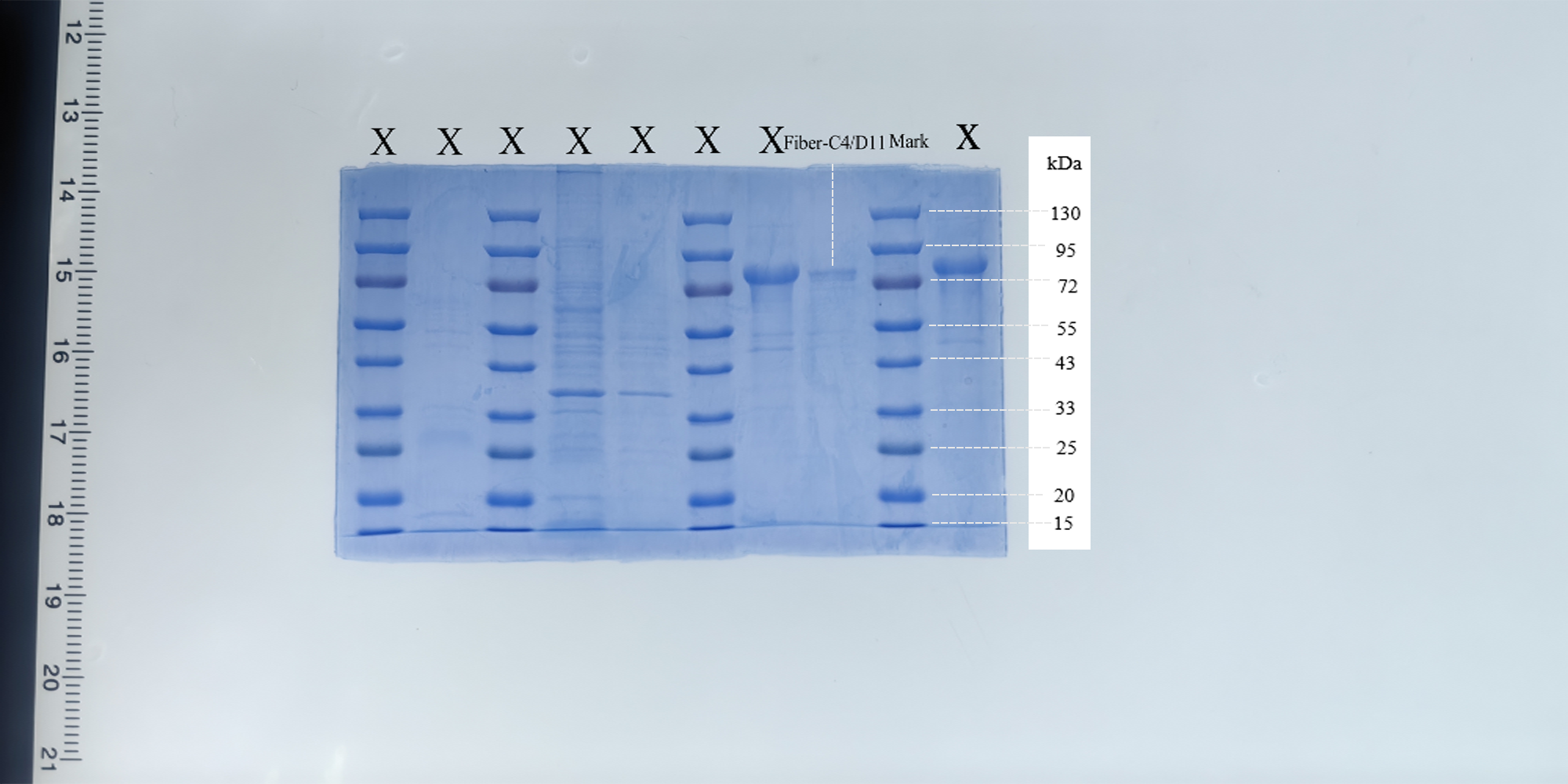

Supplement: Supplementary file 1 [file vetsci-12-00920-s001.zip › Figure S2 - Original Figure 3 -A--Fiber-C4D11.png]

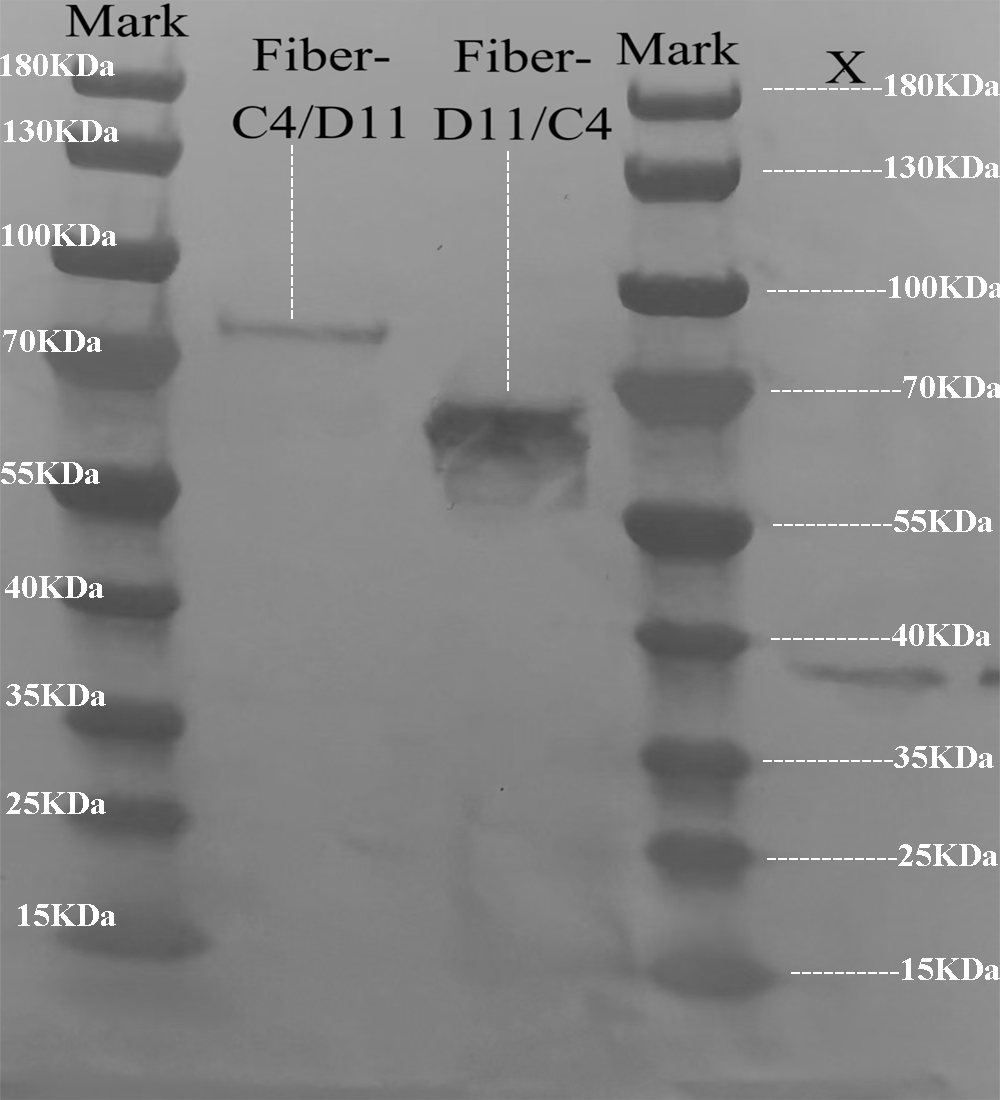

Supplement: Supplementary file 1 [file vetsci-12-00920-s001.zip › Figure S3 - Original Figure 3 B--Western Blot.png]
